# Growth Hormone-Releasing Hormone in Lung Physiology and Pulmonary Disease

**DOI:** 10.3390/cells9102331

**Published:** 2020-10-21

**Authors:** Chongxu Zhang, Tengjiao Cui, Renzhi Cai, Medhi Wangpaichitr, Mehdi Mirsaeidi, Andrew V. Schally, Robert M. Jackson

**Affiliations:** 1 Research Service, Miami VAHS, Miami, FL 33125, USA; chongxu.zhang@va.gov (C.Z.); tcui@med.miami.edu (T.C.); renzhi.cai@va.gov (R.C.); medhi.wangpaichitr@va.gov (M.W.); msm249@med.miami.edu (M.M.); andrew.schally@va.gov (A.V.S.); 2 Department of Medicine, University of Miami Miller School of Medicine, Miami, FL 33101, USA; 3 Department of Pathology and Sylvester Cancer Center, University of Miami Miller School of Medicine, Miami, FL 33101, USA

**Keywords:** idiopathic pulmonary fibrosis, growth hormone-releasing hormone, antagonists, bleomycin

## Abstract

Growth hormone-releasing hormone (GHRH) is secreted primarily from the hypothalamus, but other tissues, including the lungs, produce it locally. GHRH stimulates the release and secretion of growth hormone (GH) by the pituitary and regulates the production of GH and hepatic insulin-like growth factor-1 (IGF-1). Pituitary-type GHRH-receptors (GHRH-R) are expressed in human lungs, indicating that GHRH or GH could participate in lung development, growth, and repair. GHRH-R antagonists (i.e., synthetic peptides), which we have tested in various models, exert growth-inhibitory effects in lung cancer cells in vitro and in vivo in addition to having anti-inflammatory, anti-oxidative, and pro-apoptotic effects. One antagonist of the GHRH-R used in recent studies reviewed here, MIA-602, lessens both inflammation and fibrosis in a mouse model of bleomycin lung injury. GHRH and its peptide agonists regulate the proliferation of fibroblasts through the modulation of extracellular signal-regulated kinase (ERK) and Akt pathways. In addition to downregulating GH and IGF-1, GHRH-R antagonist MIA-602 inhibits signaling pathways relevant to inflammation, including p21-activated kinase 1-signal transducer and activator of transcription 3/nuclear factor-kappa B (PAK1-STAT3/NF-κB and ERK). MIA-602 induces fibroblast apoptosis in a dose-dependent manner, which is an effect that is likely important in antifibrotic actions. Taken together, the novel data reviewed here show that GHRH is an important peptide that participates in lung homeostasis, inflammation, wound healing, and cancer; and GHRH-R antagonists may have therapeutic potential in lung diseases.

## 1. Introduction

The goal of this review is to present and critically evaluate new findings regarding growth hormone-releasing hormone (GHRH) and its actions in the settings of lung inflammation, fibrosis, and cancer. The essential, unanswered question we address is whether GHRH, as revealed by synthetic peptide probes that activate or inhibit its receptor, plays key roles in lung pathophysiology that are distinct from its effects on growth and metabolism. It provides background on the physiology of GHRH in the lung, which was elucidated using recently developed GHRH receptor peptide agonists and antagonists as mechanistic probes. While we have used these peptides to identify effects likely attributable to GHRH, we will clearly distinguish these from the established effects of GHRH itself. Animal and cellular models of pulmonary fibrosis, lung cancer, and sarcoid have been developed and exploited to investigate the effects of GHRH-receptors (GHRH-R) inhibition or activation in lung pathophysiology.

We initially present data regarding the physiologic roles of GHRH (and by implication the effects of growth hormone (GH); then, we describe cellular and animal models used to investigate mechanisms of GHRH actions, including effects on gene expression, mitochondrial respiration, and intracellular signaling. Finally, we anticipate future directions for research and describe possible clinical applications of these approaches using synthetic peptides.

To explore GHRH actions, various peptide agonists and antagonists of the receptor have been synthesized in our laboratories [1,2,3]. For example, a GHRH-R antagonist (MIA-602) with the amino acid sequence PhAc–Ada–Tyr–DArg–Asp–Ala–Ile–5FPhe–Thr–Ala–Har–Tyr(Me)–His–Orn–Val–Leu–Abu–Gln–Leu–Ser–Ala–His–Orn–Leu–Leu–Gln–Asp–Ile–Nle–D–Arg–Ha–NH_2_ has been synthesized by solid phase methods and purified by HPLC. This antagonist and related GHRH agonists (e.g., MR-409) that bind to GHRH-R have been useful in revealing the physiologic and systemic activities of GHRH distinct from its hormonal effects on the pituitary and the GH/insulin-like growth factor-1 (IGF-1) pathway.

The preclinical evaluation of new GHRH analogs of the “Miami” (MIA) series with increased inhibitory potency is well underway. In the synthesis of these analogs, the following substitutions were conserved: D-Arg^2^, Har^9^, Abu^15^, and Nle^27^. The replacement of Lys^12^ and Lys^21^ with Orn increased resistance to enzymatic degradation. The substitution of Arg at positions 11 and 20 by His were conserved. We incorporated pentafluoro-Phe at position 6, Tyr (Me) at position 10, and ω-amino acids at the N-terminus of some analogs. Evaluation of the activity of such analogs on GH release was done in vitro on rat pituitaries and in vivo in male rats. Receptor binding affinities were measured in vitro by competitive binding analysis. The inhibitory activity of analogs on proliferation in vitro was initially tested in several human cancer cell lines such as endometrial adenocarcinoma, colorectal adenocarcinoma, and prostatic carcinoma. Several cell lines were engrafted into nude mice treated subcutaneously with GHRH antagonists at doses of 1–5 μg/day. Analogs MIA-602, MIA-604, MIA-610, and MIA-690 showed high binding affinities to the receptor, which is significantly greater than GHRH(1-29)NH_2_ itself. The treatment of tumor cells with 5 μM MIA-602 or MIA-690 decreased proliferation by 40–80%. Thus, GHRH analogs of the MIA series suppress cellular proliferation but have relatively weak endocrine GH inhibitory activity. The suppression of cellular growth likely could be induced by the downregulation of GHRH receptors levels [3].

## 2. Physiological Functions of GHRH

GHRH is a 44-amino acid peptide secreted primarily by the hypothalamus, but various other tissues including the lungs produce it locally [4]. Data reviewed here illustrate the physiologic role of GHRH per se in the lung, which is independent of effects of GHRH-R agonists or antagonists that we describe later. GHRH stimulates the release and secretion of growth hormone (GH) by the pituitary. It stimulates the production of insulin-like growth factor 1 (IGF-1) through the pituitary GH/hepatic IGF-1 axis. GHRH belongs to a peptide family that includes glucagon, secretin, vasoactive intestinal peptide, and pituitary adenylate cyclase-activating peptide [4,5]. The amino terminal sequence of 29 amino acids retains the full biological activity of GHRH. GHRH binds to its receptor on pituitary somatotrophs and activates the synthesis and secretion of growth hormone (GH). GHRH peptide and GHRH-R are expressed in normal extra-pituitary tissues, including tumors, cancer cell lines, and immune cells. A truncated but functional splice variant (SV1) of GHRH-R is found in tumors, pituitary, and peripheral tissues including the lung, implying a physiological role unrelated to its endocrine function.

The existence of a GH-releasing factor in hypothalamic extracts was shown over fifty years ago. Pancreatic tumors can produce GHRH resulting in acromegaly; this observation was made possible by the isolation and characterization of GHRH [6]. The structure of pancreatic GHRH is identical to the hypothalamic peptide. GH acts directly on receptors in peripheral tissues, but in liver, it stimulates the production of insulin-like growth factor 1 (IGF-1), which is a growth-promoting mitogen for human lung fibroblasts [7]. The maintenance of a neuroendocrine axis comprised of GHRH–GH–IGF-1 is the major endocrine function of GHRH. In pituitary and other cells, the effects of GHRH are mediated by binding to specific membrane receptors that belong to the seven-transmembrane class of G-protein coupled receptors. After the binding of GHRH to the GHRH-R of somatotrophs, second messengers include adenylate cyclase–cAMP–protein kinase A, Ca^2+^–calmodulin, inositol phosphate–diacylglycerol–protein kinase C, and arachidonic acid pathways that result in GH secretion. GHRH-R expression was originally thought to be restricted to pituitary cells due to the tissue-specific expression of transcription factor Pit-1, which plays an essential role in the differentiation of somatotrophs. Alternately spliced variants of GHRH-R such as SV1 have been detected in tissues including the human lung, and some act independently of ligand binding. Therefore, GHRH may have diverse, downstream, paracrine effects.

The enlargement of visceral organs such as the lungs, heart, and kidneys is a cardinal manifestation of acromegaly. Since increased lung volume measured by pulmonary function testing in patients with acromegaly is not related to hyperinflation or to increased inspiratory muscle strength, it is evident that the excess of GH in acromegaly induces the significant growth of adult human lungs [8]. Ectopic acromegaly is rare, and since the discovery of GHRH, few cases have been reported. Tumors secreting GHRH are typically neuroendocrine, mainly of pancreatic or bronchial origin. Patients who present with acromegaly, whose features include enlargement of the lungs, are those of a somatotropic adenoma. GHRH concentration in plasma is specific for diagnosis at a threshold of 250 to 300 ng/L. Somatostatin analogs therapeutically decrease GH secretion and inhibit GHRH secretion.

## 3. GHRH in the Lung

**Figure 1:**
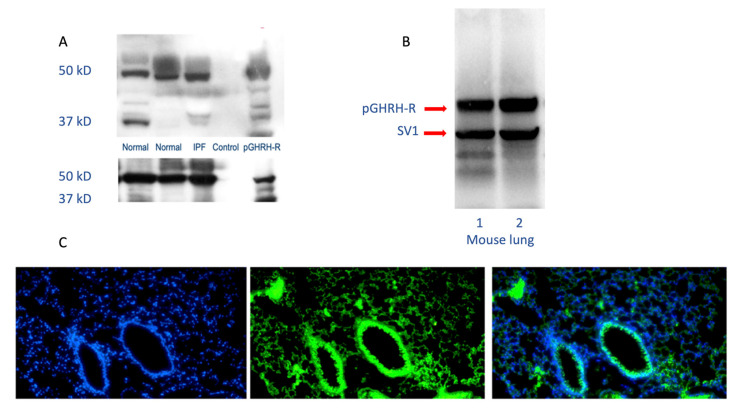
Human and mouse lung westerns (upper panels **A** and **B**) and immunofluorescence staining (lower panel **C**) demonstrate growth hormone-releasing hormone-receptor (GHRH-R) protein. As shown in the upper panels, Western blotting confirms the presence of pituitary-type GHRH-R (pGHRH-R) and splice variant (SV1) in both normal and IPF human lung tissues (upper left panel). Likewise, the pGHRH-R is abundant in lung tissue protein from normal C57BL/6J mice (upper right panel). GHRH-R was detected using a rabbit polyclonal IgG primary antibody (Origene Technologies, Inc., Rockville, MD, USA). As shown in the lower panel, immunofluorescent staining for GHRH-R protein demonstrates prominent expression of the GHRH-R protein in the bronchial epithelium, as well as in alveolar parenchymal cells. (Left, DAPI staining; middle, immunofluorescent antibody to GHRH-R; right, merged images).

The GHRH-R modulates activities of key intracellular signaling pathways involved in lung growth, inflammation, and remodeling, demonstrating that GHRH (or GH itself) could participate in lung development, growth, and repair [9,10]. GHRH-R and a splice variant of the receptor are widely distributed in rat tissues other than the hypothalamus and pituitary. GHRH-R was initially detected in rat lung tissue, using RT-PCR after RNA extraction from a whole lung [11]. The GHRH gene is expressed in many lung cells, including alveolar type 2 cells, club cells, and fibroblasts. Higher levels of gene expression are found in lymphocytes and dendritic cells [12]. GHRH-R has been identified as important in the survival of several lung cancer cell lines. Interestingly, both carcinoid tumors and small cell lung cancers produce and release GHRH into the circulation, confirming its presence in lung neuroendocrine cells [6]. As shown in Figure 1, the GHRH-R protein is clearly expressed in normal and diseased human lungs and in normal mouse lungs.

Since studies in diverse systems demonstrate GHRH-R in lung cells, and since GHRH-R antagonist modulates inflammation, fibrosis, and lung cancer, GHRH has certain biological importance in the lung and appears to participate in physiologic processes beyond lung injury and repair. Its effects on lung cellular proliferation (e.g., in tumors) are strongly implied by the inhibitory activities of GHRH-R antagonist peptides. Through paracrine signaling, lung cells respond to locally produced GHRH with a variety of effects on proliferation, metabolism, and inflammation [13,14].

### 3.1. Signaling Pathways

Several peptide GHRH-R agonists and antagonists have been developed in the laboratory; we have utilized some of these to identify specific pathways and their effects regarding the actions of GHRH in lung inflammation, fibrosis, and cancer, as we describe in the section below. Many GHRH-sensitive signaling pathways operate in the lung. These link lung inflammation, mitochondrial function, apoptosis, and fibrosis after injury with the effects of GHRH. Pathways relevant to lung inflammation, fibrosis, and cancer and the effects of GHRH-R antagonists are summarized in Table 1.

For example, A549 lung epithelial cells, derived from a broncho-avleolar cell tumor, express GHRH-R protein. These lung epithelial cells respond to inhibition of the GHRH-R by the activation of 5′ adenosine monophosphate-activated protein kinase (AMPK) and glycogen synthase kinase 3 B (GSK3B) pathways. GHRH-R antagonist also inhibits protein kinase B (Akt) and mammalian target of rapamycin (mTOR) pathways controlling growth in this cell line, facilitating apoptosis [15]. Thus, GHRH-R antagonists regulate the AMPK pathway and growth in lung cells. Pulmonary artery endothelial cells likewise respond to inhibition of the GHRH-R by the downregulation of extracellular signal related kinase (ERK1/2) and Janus kinase-signal transducer and activator of transcription (JAK2/STAT3) pathways, which are implicated in both lung inflammation and apoptosis [18,19].

**Figure 2:**
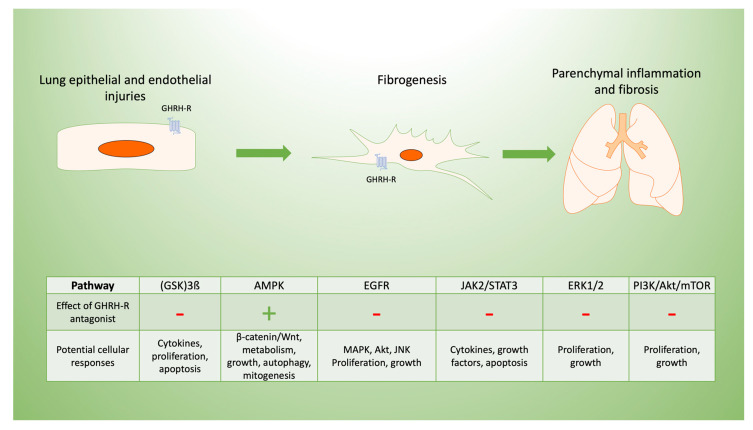
Potential mechanisms of GHRH-R antagonist in lung inflammation and fibrosis. Pulmonary fibrosis typically results from cellular injuries that may be followed by inflammation and progressive fibrosis. GHRH-R is present in lung tissue, and GHRH has effects on lung cellular functions potentially mediated by diverse signaling pathways. These are involved in the lung’s response to inflammation and resulting fibrosis, which may be in response to epithelial injuries as shown diagrammatically above. GHRH-antagonist peptides maintain endothelial barrier function disrupted by inflammation, as they downregulate extracellular signal related kinase (ERK1/2) and Janus kinase-signal transducer and activator of transcription (JAK2/STAT3). In lung epithelial cells, GHRH-R antagonist peptides activate adenosine monophosphate-activated protein kinase (AMPK) and glycogen synthase kinase 3 B (GSK3B) while inhibiting Akt/mammalian target of rapamycin (mTOR) and modulating cellular injury. Similarly, GHRH-R antagonist has anti-proliferative effects in several lung cancer cell lines, which are mediated by epidermal growth factor receptor (EGFR) pathways.

Specific signaling pathways modulated by GHRH may be targeted to limit inflammation and post-inflammatory fibrosis, as we summarize in Figure 2. For example, the inhibition of p38 MAPK phosphorylation in C57Bl/6 mice decreases renal fibrosis [20]. The constitutive expression of Akt renders lung tissue susceptible to pulmonary fibrosis [21], in part due to its effects on cellular proliferation [22]. Both pathways are modulated by inhibition of the GHRH-R, as described below.

Human fibroblasts express GHRH receptors, which mediate a regulatory effect on proliferation through ERK and Akt signaling. These two signaling cascades are involved in proliferation and apoptosis. Cui and others from our laboratory have reported that when skin wounds in mice are exposed to GHRH agonist, fibroblasts proliferate, and repair of the epithelium is accelerated [1]. Then, fibroblast proliferation induced by a GHRH agonist could be inhibited by blocking ERK. The GHRH receptor is G-protein coupled; ligand binding to GHRH receptor directly leads to cyclic adenosine monophosphate (cAMP)-dependent activation of ERK and Akt pathways. GHRH agonists MR-409 and MR-502 both increase cellular cAMP levels and support cellular growth.

GHRH also stimulates the expression of α-smooth muscle actin (αSMA) through phosphatidylinositol 3-kinase/protein kinase B (PI3K/Akt) signaling, which stimulates contractile activity [23]. ERK activity modulates cellular proliferation by enhancing apoptosis, autophagy, and senescence [19], and GHRH-R antagonist peptides may regulate these activities through downregulation of the ERK pathway.

Microvascular endothelial cells express both the pituitary-type GHRH receptor and SV1. Endothelial cells are responsible for angiogenesis, which is a critical event in wound healing. Uddin and others have shown that GHRH-R peptides suppress the activation of MLC2, ERK1/2, and JAK2/STAT3 and increase p53 and pAMPK, which support the endothelial permeability barrier [16]. GHRH produced by fibroblasts regulates the activities of other cells involved in wound healing in a paracrine fashion. Overall, activation of the GHRH-R provides pro-inflammatory and pro-fibrotic signals, while GHRH-R antagonists modulate inflammation and fibrosis, as described below.

### 3.2. Fibrogenesis

Transforming growth factor-beta (TGF-β), Wingless and Int-1 (Wnt), hedgehog, Notch, and fibroblast growth factor (FGF) signaling pathways are implicated in regulating lung morphology during development and participate in fibrosis [24]. Wnt/β-catenin signaling is further essential to the regulation of myofibroblast differentiation of mesenchymal cells in the lung and participates in the development of idiopathic pulmonary fibrosis (IPF) [25]. The Wnt/B-catenin pathway is expressed in lung epithelial cells and regulates epithelial and mesenchymal cell interactions during the development of fibrosis. Wnt4 is upregulated by GH and leads to the activation of ERK1 and STAT3, modulating cell growth and survival [26]. Many such pathways are activated during injury and repair and have been linked to post inflammatory fibrosis [27]. Persistent activation, e.g., by TGF-β, can result in lung pathologies, including IPF [28].

The v-AKI murine thymoma viral oncogene/protein kinase B (Akt/PKB) is a serine/threonine-specific kinase involved in apoptosis and proliferation. Akt/PKB regulates cellular survival and metabolism by regulating downstream effectors such as nuclear factor kappa B (NF-κB) and Bcl-2 family proteins in human cancer cells [29,30]. Cells respond through Akt/PKB to a variety of cytokines, G protein coupled receptor ligands, and growth factors, making it a potential target to modulate fibrosis. Consistent with this mechanism, the suppression of Akt and mTOR by GHRH-R antagonist treatment inhibits the growth of A549 epithelial cells in culture, and in contrast, GHRH agonists stimulate the Akt pathway.

In addition to its effects on GH and IGF-1, synthetic GHRH-R antagonist MIA-602 inhibits p21-activated kinase 1-signal transducer and activator of transcription 3/nuclear factor-kappa B (PAK1-STAT3/NF-κB), which is consistent with its role in modulating inflammatory and fibrotic processes [31]. GHRH-R antagonist activity is in part modulated by p53 and p21, which suppress NF-KB and inducible nitric oxide synthase (iNOS) while facilitating apoptosis [32,33].

Fibroblast growth factor-2 (FGF-2) is expressed in the epithelium, vascular endothelium, smooth muscle and epithelial basement membrane; and, an increased expression of FGF-2 occurs in lungs from patients with IPF. FGF-receptor regulates fibroblast apoptosis through Akt and focal adhesion kinase (FAK) related to TGF-β activation [13]. Several pro-fibrotic pathways, including Akt and ERK, are inhibited by GHRH-R antagonists including MIA-602, demonstrating that GHRH plays a key role in modulating lung fibrosis following inflammation by blocking these pathways [1,34].

PI3K-Akt signaling inhibits apoptosis and regulates cell growth, survival, and proliferation. Receptor activation recruits PI3K to the inner cell membrane through phosphorylated tyrosine kinases, activated proto-oncogene protein p21 (RAS), or G protein beta and gamma subunits [29]. Akt plays important roles in response to growth factors to regulate metabolism, growth, apoptosis, and survival. In response to injury, sustained PI3K activation worsens lung fibrosis due to bleomycin [23]. The PI3K–Akt pathway is involved in the pathogenesis of fibrosis and regulates epithelial to mesenchymal transition.

MIA-602 has effects on lung fibroblast signal transduction, including anti-apoptotic Akt/protein kinase B (PKB) and pro-growth extracellular signal-regulated kinase (ERK), which are two of the key pathways controlling fibroblast survival and proliferation. Bleomycin increased Akt phosphorylation and concomitantly decreased ERK phosphorylation after an in vitro incubation of lung fibroblasts. In contrast, MIA-602 reduced Akt phosphorylation due to bleomycin and restored ERK activation in lung fibroblasts treated with bleomycin. Thus, inhibition of the GHRH-R appeared to prevent activation of the PI3K–Akt pathway caused by bleomycin, which is consistent with the observed reduction of inflammation and fibrosis [35].

TGF-β and bFGF regulate apoptosis in granulation tissue fibroblasts while inhibiting Akt [28]. The inhibition of GHRH-R by MIA-602 decreases Akt activation after bleomycin and leads to fibroblast apoptosis. GHRH-R antagonist causes apoptosis in epithelial cells through the suppression of mitogen-activated protein kinases (MAPK) and inhibits epithelial to mesenchymal transition, so limiting fibrosis. GHRH inhibitors also act to reduce fibrosis through the suppression of MAPK and p53 [32].

## 4. GHRH and Cellular Respiration

GHRH-R antagonist peptides influence fibroblast respiration and apoptosis in ways that impact fibrogenesis. MIA-602 increased basal oxygen consumption and maximal, uncoupled respiration of normal mouse lung fibroblasts; it also increased both basal respiration and spare respiratory capacity [17]. The maintenance of cellular respiration is evidently supported by MIA-602, and enhanced mitochondrial function would maintain fibroblast apoptotic capacity, which is consistent with the observed and potentially beneficial inhibition of fibrosis.

Mitochondrial function and reactive oxygen species (ROS) production are functionally linked to cell death. In the intrinsic pathway, apoptotic proteins cause mitochondrial membrane damage, depolarization, pore formation, and the release of calcium and cytochrome C. DNA damage, which is detectable in the alveolar epithelium of humans with IPF and in rodent models of lung fibrosis, triggers p53-regulated intrinsic apoptosis. Preventing mitochondrial damage in lung injury models mitigates fibrosis. The inhibition of pro-apoptotic protein Bax attenuates bleomycin-induced apoptosis, as well attenuates mouse lung fibrosis. The maintenance of fibroblast apoptosis by the enhancement of mitochondrial function, as shown with MIA-602, would limit mesenchymal proliferation and fibrosis [36,37].

## 5. GHRH and Oxidative Stress

### 5.1. Oxidative Metabolism

Interestingly, GH itself induces oxidative stress by augmenting the respiratory burst in granulocytes and macrophages; it is apparently involved in the inflammatory response as demonstrated in several model systems we have used [10,38,39,40]. GH and the GH-dependent growth promoting peptide, IGF-I, are both signals for priming polymorphonuclear neutrophils (PMN) to secrete superoxide anion (O_2_^-^). A specific antibody to GH eliminated priming. A monoclonal antibody directed against the human IGF-I receptor blocked the secretion of O_2_^-^ by human PMN caused by IGF-I but not GH, indicating that neutrophil priming induced by GH was not mediated by the extracellular release of IGF-I. The pituitary hormone (GH), as well as growth promoting IGF-I, are involved in modulating oxidative stress. GH and IGF-I are synthesized by leukocytes, so these data support the view that both proteins act in a paracrine fashion to prime neutrophils for an enhanced respiratory burst.

### 5.2. Antioxidant Effects

The synthetic GHRH-R antagonists described herein exert growth-inhibitory effects both in vitro and in vivo, in addition to having potential anti-inflammatory and anti-oxidative effects. GHRH-R antagonists inhibit inducible nitric oxide synthase (iNOS) and cyclooxygenase-2 (COX-2) (31, 32), decreasing inflammation in lung tissue consistent with our observations in mice treated with bleomycin described below. MIA-602 also decreased the nitrotyrosine (NT) content of lung fibroblasts incubated in vitro with bleomycin, indicating that it had anti-oxidative and anti-nitrosative effects that limited peroxynitrite formation [35].

## 6. GHRH Receptors in Inflammation and Fibrosis

### 6.1. Inhibition of Inflammation and Fibrosis

GHRH-R antagonist MIA-602 limits inflammation and fibrosis in a model of intraperitoneal, bleomycin-induced lung fibrosis. MIA-602 effectively decreased inflammation (reported as histopathological scores) due to bleomycin, compared to mice receiving vehicle [17].

MIA-602 also reduced fibrosis in lungs of bleomycin-treated mice. Hydroxyproline (HP) content increased significantly after 28 days in mice treated with bleomycin; no significant increase in HP content occurred in lungs of mice also treated with the GHRH-R antagonist MIA-602.

### 6.2. Effects of GHRH-R Antagonist on Gene Expression

We assessed mechanisms by which MIA-602 could modulate inflammation or fibrosis by sequencing RNA libraries (RNA-seq) from mouse lungs treated with bleomycin and MIA-602 or vehicle and from normal mouse lung fibroblasts [41]. We completed gene set enrichment and pathway analyses based on the functional annotation of differentially expressed genes in mouse lung tissue and fibroblasts to find if differentially expressed genes were associated with any observed effects of MIA-602.

The downregulation of T-cell receptor complex genes (CD3E, CD3G, CD4, and CD8A) had high associations in pathway analyses. T-cell receptors and costimulatory molecules are required for the activation of T-cells and in the development of inflammation-driven lung fibrosis [42,43]. MIA-602 appears to play an important role in the homeostasis of lung tissue by the modification of T-cell signaling, while reducing inflammation and fibrosis.

After bleomycin was administered, genes related to extracellular matrix and Wnt signaling were upregulated in lung (absolute fold change >1.5 and false discovery rate 0.01), which is consistent with known effects of bleomycin in fostering fibrosis. Several genes were downregulated by bleomycin, including those related to lung morphogenesis and development, extracellular matrix organization, and alveolar septal development.

In lungs from mice treated with both bleomycin and MIA-602, genes related to chemotaxis, IL-1, chemokines, and the regulation of inflammation and ERK cascade were upregulated. Multiple genes related to immune response and T-cell functions were downregulated, which is consistent with the anti-inflammatory effect of MIA-602.

## 7. GHRH and Pulmonary Sarcoid

We have recently described novel in vitro and in vivo granuloma models to investigate inflammation in sarcoidosis [44,45]. Sarcoidosis is a multi-organ, granulomatous disease that affects the lungs and is associated with significant morbidity and mortality. It triggers the recruitment of Th1 helper cells and a later phase in which macrophages produce granulomas. We have assessed the anti-inflammatory effects of GHRH-R antagonist MIA-602 in sarcoid-like granulomas. To accomplish this, we established a granuloma model using peripheral blood mononuclear cells (PBMC) from patients with sarcoidosis. PBMC develop into granulomas when exposed to microparticles from *Mycobacterium abscessus*. The granulomas produced significant increases in several pro-inflammatory cytokines compared to the unchallenged PBMC. MIA-602 reduced in vitro cytokine production by granulomas, demonstrating suppression of the inflammatory response in response to GHRH-R inhibition. A significant decrease in the release of inflammatory cytokines IL-2, IL-12, and IL-17A occurred in granulomas incubated with MIA-602. CD45+ and CD68+ cells were decreased in the experimental granulomas. Similarly, mice challenged with *M. abscessus* microparticles also developed a granulomatous inflammatory reaction in lungs. Flow cytometric analysis showed that MIA-602 significantly reduced the population of CD68+ cells (monocytes/macrophages) in the lungs of mice with granulomatous inflammation.

Bcl-xL/Bak dimer levels increased in granulomas after treatment with MIA-602. Active caspase-3 levels increased in granulomas likely due to lymphocyte activation, further supporting the notion that the GHRH-R antagonist is involved in apoptosis.

## 8. GHRH Antagonists and Lung Cancer

### 8.1. GHRH in Lung Cancer Models

GHRH-R and its splice variant are implicated in the antitumor effects of GHRH antagonists. The expression of non-hypothalamic GHRH, GHRH-R, and SV1 has been demonstrated in tumors, showing that locally produced GHRH can function as an autocrine/paracrine growth factor. Cancer cells transfected with SV1 exhibit increased cell proliferation, suggesting that the blockade of ligand-independent activity of SV1 might lead to effective therapies.

**Figure 3:**
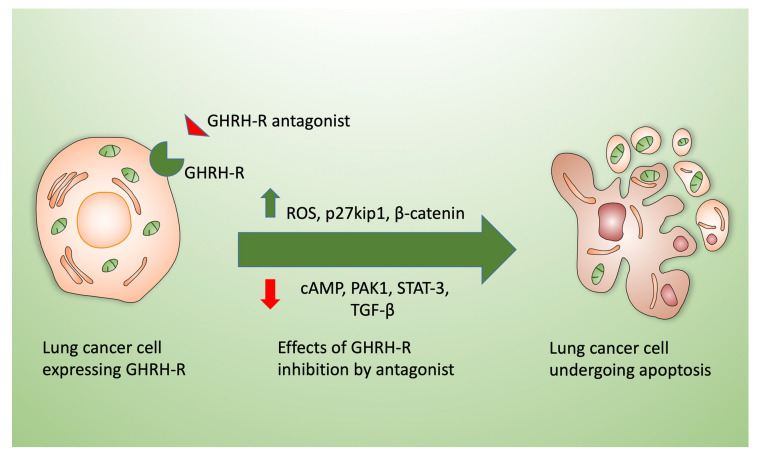
Effects of GHRH-R antagonists (e.g., MIA-602 or MIA-690) on lung cancer cells. Exposure of lung cancer cells in culture (left) to GHRH-R antagonists leads to cell death by apoptosis (right). Mechanisms triggering cell death include the enhanced production of reactive oxygen species (ROS) by the cells after antagonist treatment and the activation of p27kip1 and ß-catenin. MIA-602 and similar inhibitors decrease cellular cAMP, p21-activated kinases (PAK), p-Signal transducer and activator of transcription 3 (STAT3), and transforming growth factor-beta (TGF-β) in addition to downregulating the receptor itself and cyclins.

Recently, we have described a significant growth inhibitory effect of GHRH-R antagonists (including MIA-602 and MIA-690) in lung cancer models both in vitro and in vivo [46,47,48,49]. Other mechanisms of observed antitumor effects attributed to GHRH-R inhibition include increased P27kip1 and the downregulation of pituitary-type GHRH receptors, which are linked to the cell cycle and cAMP/cAMP response element-binding protein (CREB) signaling pathways, as shown diagrammatically in Figure 3.

GHRH-R antagonists reduce mesothelioma cell survival [46], demonstrating the ability to sensitize cells to chemotherapy, as reported in other systems by Wangpaichitr and colleagues [50]. GHRH antagonists impaired mitochondrial function and increased ROS, resulting in cell death by apoptosis. MIA-602 and MIA-690 also blunted the expression of MMP-2 and MMP-9, which are key mediators of tumor growth, metastasis, and angiogenesis [46]. Several factors could contribute to therapeutic synergism, including pro-apoptotic signaling and the inhibition of oncogenic and anti-apoptotic molecules including p12-activated kinase-1, cAMP response element binding protein, and cyclin-dependent kinases by GHRH antagonists [48,49,50].

Survival pathways, which might be downregulated by GHRH-R inhibition, are implicated in resistance to chemotherapy, including PI3K/Akt, which is a downstream component of IGF-I receptor and a major anti-apoptotic pathway [51].

Along with endocrine effects on the GH/IGF-I axis, direct mechanisms likely include blockade of the autocrine/paracrine activity of GHRH and IGF-I/II in lung cancers. GHRH antagonists suppress phospho-STAT3 in lung cancers. STAT3 signaling contributes to crosstalk between tumor and immune cells, including macrophages, CD8+ T-cells, myeloid-derived suppressor cells, and regulatory T-cells. STAT3 activation confers high Programmed death-ligand (PD-L) expression, which promotes tumor immune evasion [49]. The combination of PD-1/PD-L1 antibodies and GHRH-R antagonists is a potentially important therapeutic approach for lung cancer that bears investigation.

### 8.2. Antioxidant Effects of GHRH-R Antagonist

As reviewed above, MIA-602 and MIA-690 induce changes in mitochondrial function, resulting in elevated reactive oxygen species (ROS), which is consistent with the data reviewed above regarding the effects of MIA-602 on fibroblast respiration [17]. An increase in intracellular ROS due to chemotherapy would render cancer cells beyond their baseline ability to tolerate oxidant stress, leading to cell death. Chemotherapy-resistant lung cancers maintain higher basal levels of ROS and are susceptible to ROS-inducing chemotherapy [51]. Cisplatin-resistant (CR) lung cancer cells, regardless of the signaling pathway status, share characteristics of an increase in ROS production and metabolic reprogramming. Cisplatin inhibits thioredoxin (TRX), leading to elevated ROS levels; and, repeated exposure to cisplatin leads to lower TrxR/TRX levels and high basal ROS levels. Cisplatin-resistant cells may adapt to survive under high levels of ROS by activating other antioxidant systems, and they rely on oxidative metabolism. They are highly sensitive to glutamine deprivation. Such metabolic adaptations could be exploited and targeted by GHRH-R antagonists. Given the apparent anti-oxidative properties of MIA-602 and other GHRH-R antagonists, such findings support the potential trials of efficacy of GHRH-R antagonists in cisplatin-resistant lung tumors.

## 9. Summary and Clinical Context

GHRH-R protein is readily detectable in human and mouse lung tissue and is widely expressed in mouse lung airway epithelial and parenchymal cells. Among our prominent findings, GHRH-R antagonist MIA-602 is shown to have diverse effects on gene expression consistent with decreased inflammation and matrix production. This synthetic GHRH-R peptide antagonist decreases both lung inflammation and fibrosis after bleomycin. GHRH-R antagonist MIA-602 exerts pro-apoptotic effects on lung fibroblasts, which is mechanistically consistent with our finding that it enhances mitochondrial oxygen consumption. Likewise, GHRH-R antagonists modulate inflammation in an in vitro lung granuloma model, again demonstrating that such molecules possess anti-inflammatory properties. We have further substantiated significant growth inhibitory effects of GHRH-R antagonists on lung cancer cells, providing another avenue for preclinical evaluation of their utility.

GHRH-R binds GHRH and stimulates the paracrine production of GH that is essential for growth, development, and tissue repair. GH exerts multiple effects relevant to lung growth, function, and repair. For example, GH stimulates lung growth in acromegaly, and GHRH-R agonists increase fibroblast proliferation and accelerate wound healing. GHRH itself enhances tumor growth, and GHRH-R antagonists inhibit tumor stromal fibrosis in experimental models. Diseases characterized by fibroblast proliferation such as IPF could be downregulated by targeting specific pathways that mediate GHRH effects. Antagonism of the GHRH-R is by itself sufficient for that purpose. When an antifibrotic effect is required, apoptosis could be supported by the enhancement of fibroblast mitochondrial function by the inhibition of GHRH-R using an antagonist such as MIA-602.

A prominent role for GHRH in lung physiology and a possible therapeutic role for GHRH-R antagonists has been established in experimental systems ranging from in vitro to cell culture to animal models. Lung cancer model systems have clearly revealed possible therapeutic potentials for GHRH-R antagonists. The next logical step is to validate the safety and effectiveness of selected GHRH-R peptides in clinical trials, possibly including patients with lung injury, sarcoid, bronchogenic carcinoma, or viral pneumonia such as that due to SARS-CoV-2. Our laboratory is accordingly proceeding to prepare pharmaceutical grade GHRH-R peptides to initiate this goal.

These data establish a rationale for and demonstrate the technical feasibility of further investigating a role for GHRH in the pathogenesis of pulmonary inflammation, fibrosis, and lung cancer. They clearly suggest that MIA-602 or similar GHRH-R antagonists could be rapidly translated into useful therapeutic agents in the context of lung disease.

## 10. Patents

A.V.S., R.C., R.M.J. and M.M. are listed variously as co-inventors on United States patents regarding the use of GHRH analogs in cancer, fibrosis and sarcoid, which are assigned to the University of Miami and the Department of Veterans Affairs.

## Figures and Tables

**Table 1:** Effects of GHRH-R antagonists in lung inflammation and cancer models.

GHRH-R Antagonists	Model System	Pathways Implicated in Effects	Potential Effects	Reference
MZ-5-156	Lung cancer	AMPK ↑ Akt/mTOR ↓ GSK3β ↓	Anti-proliferative	[15]
MIA-602	Lung endothelial cells	ERK ↓ JAK2/STAT3 ↓ p53 ↑ AMPK ↑	anti-inflammatory	[16]
MIA-602	Mouse lung and fibroblasts	ERK ↑ Akt ↓	anti-inflammatory, anti-fibrotic, pro-apoptotic	[17]
